# Evolutionary path from a prisoner’s dilemma to a harmony game via hawk–dove games

**DOI:** 10.1038/s41598-026-47565-9

**Published:** 2026-04-13

**Authors:** Balázs Király, Tamás Varga, József Garay

**Affiliations:** 1 HUN-REN Centre for Energy Research, Institute of Technical Physics and Materials Science, Konkoly-Thege Miklós út 29–33, H-1121 Budapest, Hungary; 2 Bolyai Institute, University of Szeged, Aradi vértanúk tere 1, H-6720 Szeged, Hungary; 3 National Laboratory for Health Security, University of Szeged, Aradi vértanúk tere 1, H-6720 Szeged, Hungary; 4 HUN-REN Centre for Ecological Research, Institute of Evolution, Konkoly-Thege Miklós út 29–33, H-1121 Budapest, Hungary

**Keywords:** Matrix game, Trait evolution, Payoff evolution, Social dilemma, Evolutionarily stable strategy transition, Ecology, Ecology, Evolution, Mathematics and computing, Physics, Zoology

## Abstract

In most evolutionary matrix game models, the payoffs are assumed to be constant. Here, we develop a toy model of hunting in pairs in which interaction outcomes are determined not only by the behavioural strategy choice of the players but also a second, evolvable physical trait of them. We analyse the evolutionary dynamics of this model in a limiting case of the classical monomorphic framework introduced by Maynard Smith and Price characterised by rare mutations in physical trait, moderately frequent mutations in behaviour, and very fast selection in both. We show that this process can transform the evolutionarily stable strategy and, as a result, even the nature of the game played in the population: We present an illustrative numerical example in which the concurrent evolution of the behavioural and trait components of the phenotype turns a social dilemma into a harmony game via an intermediate phase of coexistence.

**Supplementary Information:**

The online version contains supplementary material available at 10.1038/s41598-026-47565-9.

## Introduction

The emergence of cooperation in social dilemma situations is undoubtedly one of the most popular topics in evolutionary game theory^1^. In a social dilemma, individual and group interests are at odds with each other. This typically manifests in the competition of selfish individuals as a risk that an individual might end up having to shoulder the cost of a joint venture alone while their partner reaps the profits.

Classical game theory^2^ advises selfish and rational players to choose their strategies according to a Nash equilibrium^3^ – a strategy profile from which no player has an incentive to unilaterally deviate –, which in social dilemma situations translates to players forgoing cooperation even though it could be, if reciprocated, more beneficial to them. Based on the iterative principle of the shadow of the future^4,5^, the dilemma persists even when the game is repeated a finite number of times. In line with the Nash equilibrium solution concept, the criterion of evolutionary stability^6^ also favours selfish, non-cooperative behaviour when a social dilemma is played in a well-mixed population.

Despite the predictions of the above theoretical considerations, numerous real-life social dilemma situations exhibit even long-lasting cooperation between participants. Consequently, most analyses of mechanisms that engender and maintain cooperation exploit “loopholes” (which mirror real conditions) that discard or bypass the basic assumptions (which typically oversimplify reality) underlying Neumann and Morgenstern’s selfish rationality and Maynard Smith and Price’s evolutionary stability concepts. Without being comprehensive, we can identify four rough, sometimes overlapping main directions in which these efforts to explain the prevalence of cooperation depart from Maynard Smith and Price’s model of Darwinian evolution.

The arguably most prominent of these directions rejects the assumption that interaction patterns are well-mixed within the population and introduces more frequent interactions between certain individuals instead. This increased interaction frequency can be attributed to a wide range of causes, including kinship and group affiliation^7^, spatial and social closeness^8^, and the coevolution of the interaction network^9–11^.

Another popular direction focuses on the dynamical aspects and considers how different metastrategies (strategies of choosing strategies in repeated games) support cooperation. This often involves reactive strategies, which derive the next choice of a player either directly or indirectly^4,7,8,12–16^ from the past choices of their opponents.

A third direction equips the players with additional options to influence outcomes besides choosing their strategy, such as voluntary participation^17^ or the ability to punish non-cooperative players at an extra cost^18,19^.

The fourth direction revises another key assumption of Maynard Smith and Price’s model in that it explicitly couples the possible interaction outcomes to the frequencies of the strategies. This approach can, for example, be used to model feedback effects between population density and fitness (often mediated by the environment)^20–28^ or non-uniform interaction rates^29–33^.

In the present article, we demonstrate that a fifth, markedly different direction is not only capable of avoiding or resolving but even abolishing social dilemma situations. While the above mentioned approaches incorporate a wide array of features from real social and biological systems, they all maintain the assumption that the payoffs of two individuals using fixed strategies remain unchanged. In contrast, we base our approach on the following observation: If two competing animals of the same species decide to fight for a resource, whether it be a physical or ritualized contest, and the individuals differ in a trait (strength, speed, endurance, hormone level, fang or bone strength, antler or tail size, etc.) that decides the outcome, then this trait will be subject to evolution, and its resulting change will affect the payoffs of the contest. Therefore, we consider the coevolution of payoffs^34–36^ that depend on an otherwise independent trait component of the phenotype^37^ in a framework consistent with Maynard Smith and Price’s model. We determine the evolutionary trajectory in the limit of small, rare mutations in physical trait, faster mutation in behaviour, and very fast selection in both, and show in a toy model of hunting in pairs that the concurrent evolution of these two components can transform the nature of the game played in the population.

## Results

### The model

In order to show that the evolution of interactions can catalyse cooperation in asexual, well-mixed, frequency-dependent matrix games, we constructed a theoretical behavioural ecological model. Although our model is inspired by group hunting in lions, we do not aim to provide a realistic description of how social structures develop in lion or other group hunting animal populations. Instead, we focus on exploring the capabilities of matrix games with evolving interactions as a modelling tool. As a proof of concept, our following analysis restricts itself to describing the general model framework and discussing an arbitrary, illustrative example. We believe that a technical, exhaustive exploration of our stylized model would offer no further insight about its salient features.

The lion is the only cat that lives in groups, so it is reasonable to assume that its ancestors were solitary hunters too^38^. Hunting in groups allows lions to kill large prey animals that live in herds such as blue wildebeest, buffalo, giraffes, and elephants^39–42^. Lions are one of the largest cats, only tigers weigh more^43^. Predator size is a determining factor in both the size of the prey the predator can take on^44,45^ and the amount of food the predator needs to consume to survive and reproduce^46^.

For cooperative hunting to emerge, individuals must first form at least one successful hunting pair, so we consider theoretical ecological scenarios in which two otherwise independent predators randomly happen to hunt for the same type of prey. We also assume, that the prey animals form herds for protection against predators^47,48^. The herds contain younger and smaller, therefore easier to catch, and larger, therefore stronger and riskier to catch, specimens too.

The predators hunt the herd using one of two pure strategies: They either stalk (*S*), that is, approach in hiding and ambush, or chase (*C*), that is, openly pursue and attack. Regardless of its chosen strategy, a successful solitary predator captures a prey of mass $k(m_1)$ determined by its own size $m_1$, and then proceeds to consume as much of the flesh as it can up to its appetite $b(m_1)$, which also depends on the $m_1$ size of the predator. Ultimately, the actual intake of the predator is the lower of the two quantities,

1 $$\begin{aligned} f(m_1)=\textrm{min}\{k(m_1),b(m_1)\}. \end{aligned}$$

We make the following assumptions about the outcomes when the predators hunt in pairs: Their probabilities of success depend on their chosen strategies, but aside from this correlation, they miss or capture their prey independently of each other. That is, when both predators stalk, they each succeed with a probability of $w_{SS}$; when one of them stalks and the other chases, the stalker and chaser respectively capture their prey with probabilities of $w_{SC}$ and $w_{CS}$; and when they both chase, they each manage to kill with a probability of $w_{CC}$.

We assume that a predator that has successfully captured its prey does not guard its

2 $$\begin{aligned} r(m_1)=k(m_1)-f(m_1)=\textrm{max}\{k(m_1)-b(m_1),0\} \end{aligned}$$

leftovers of the carcass, creating a scavenging opportunity for an unsuccessful partner. If only one, $m_2$-sized predator manages to kill its prey, then the other, unsuccessful, $m_1$-sized predator’s intake is

3 $$\begin{aligned} f_R(m_1|m_2)=\textrm{min}\{b(m_1),r(m_2)\}. \end{aligned}$$

We further assume that a successful predator does not stoop to scavenging, not even if it remains unsatisfied after consuming its prey.

Based on the above considerations, we can now calculate the expected game theoretic payoffs of the two predators for all four pure strategy pairings.

When both players stalk, then they each can gain access to food in two ways: They either hunt successfully – which happens with a probability of $w_{SS}$ –, and thus feed according to the $f(m_1)$ function; or after suffering failure – which occurs with a probability of $(1-w_{SS})$ –, they scavenge $f_R (m_1|m_2)$ from their partner’s leftovers – which only exist if the partner was successful with an independent $w_{SS}$ probability and the partner’s appetite was satisfied before consuming all of the meat. Regardless of the outcome, the payoff is reduced by the expected $c_S(m_1)$ cost of stalking, which arises from the energy expenditure and the risk of injury associated with this form of hunting. In summary, the expected payoff of the stalker of size $m_1$ can be written in this situation as

4 $$\begin{aligned} a_{SS}(m_1,m_2)=w_{SS}f(m_1)+(1-w_{SS})w_{SS}f_R(m_1|m_2)-c_S(m_1). \end{aligned}$$

When one of the predators chases and the other stalks, the chaser captures its prey with a probability of $w_{CS}$, while its stalker partner kills with an independent probability of $w_{SC}$. If successful itself, the chaser consumes an $f(m_1)$ amount of meat, regardless of how its partner fared. The chaser can still find food if it fails, but only if its stalker partner succeeds, in which case the chaser’s intake becomes $f_R(m_1|m_2)$. Chasing prey is expected to entail a cost of $c_C(m_1)$. Thus, the payoff function of an $m_1$-sized chaser whose $m_2$-sized partner stalks is

5 $$\begin{aligned} a_{CS}(m_1,m_2)=w_{CS}f(m_1)+(1-w_{CS})w_{SC}f_R(m_1|m_2)-c_C(m_1). \end{aligned}$$

By the same argument as above, we find that choosing to stalk while the other predator chases promises a payoff of

6 $$\begin{aligned} a_{SC}(m_1,m_2)=w_{SC}f(m_1)+(1-w_{SC})w_{CS}f_R(m_1|m_2)-c_S(m_1 ); \end{aligned}$$

and similarly, the payoff of a chaser hunting with another chaser is given by

7 $$\begin{aligned} a_{CC}(m_1,m_2)=w_{CC}f(m_1)+(1-w_{CC})w_{CC}f_R(m_1|m_2)-c_C(m_1). \end{aligned}$$

The phenotype of a predator is characterized by two attributes, one behavioural and one physical. The former describes the propensity of the predator for choosing to stalk rather than chase its prey. It can be represented by a mixed strategy of the game introduced above, which we will denote by $\textbf{p}_1=(p_1,1-p_1)$, where $p_1$ is the probability that the predator chooses to stalk in any given joint hunt with another predator. The latter trait is simply the $m_1$ measure of the size of the predator used above.

Following in the footsteps of Maynard Smith and Price^6^, we consider the evolutionary stability of these two-dimensional phenotypes in a well-mixed, asexually reproducing, monomorphic population perturbed by rare mutations. When a $(\textbf{p}_M,m_M)$ mutant phenotype emerges in a small $\varepsilon$ relative frequency in a resident population of homogeneous $(\textbf{p}_R,m_R)$ phenotype, the fitness of the two groups as defined by the average payoff of the specimens that belong to them reads

8 $$\begin{aligned} W_R(\varepsilon )&=(1-\varepsilon )\textbf{p}_RA\textbf{p}_R+\varepsilon \textbf{p}_RB\textbf{p}_M,\end{aligned}$$

9 $$\begin{aligned} W_M(\varepsilon )&=(1-\varepsilon )\textbf{p}_MC\textbf{p}_R+\varepsilon \textbf{p}_MD\textbf{p}_M. \end{aligned}$$

The entries of the four payoff matrices in these expressions are the four payoff functions defined above evaluated at the appropriate combinations of the physical trait variables of the two phenotypes^37^, to wit:

10 $$\begin{aligned} {A}&=\begin{pmatrix}a_{SS}(m_R,m_R) & a_{SC}(m_R,m_R) \\ a_{CS}(m_R,m_R) & a_{CC}(m_R,m_R) \end{pmatrix}, {B}=\begin{pmatrix}a_{SS}(m_R,m_M) & a_{SC}(m_R,m_M) \\ a_{CS}(m_R,m_M) & a_{CC}(m_R,m_M) \end{pmatrix},\end{aligned}$$

11 $$\begin{aligned} {C}&=\begin{pmatrix}a_{SS}(m_M,m_R) & a_{SC}(m_M,m_R) \\ a_{CS}(m_M,m_R) & a_{CC}(m_M,m_R) \end{pmatrix}, {D}=\begin{pmatrix}a_{SS}(m_M,m_M) & a_{SC}(m_M,m_M) \\ a_{CS}(m_M,m_M) & a_{CC}(m_M,m_M) \end{pmatrix}. \end{aligned}$$

The resident phenotype is called an evolutionarily stable phenotype (ESP) if it cannot successfully be invaded by another phenotype, that is, if its fitness is at least as high as that of any competing distinct mutant phenotype that might emerge or, more precisely, if for any $(\textbf{p}_M,m_M )\ne (\textbf{p}_R,m_R)$ phenotype and any small enough, non-vanishing $\varepsilon$ relative frequency $W_R(\varepsilon )>W_M(\varepsilon )$ holds^6,37^. Conversely, if a resident phenotype is not evolutionarily stable, it can and eventually will be replaced by a more successful mutant.

### The dynamics

In what follows, let us restrict our analysis to the dynamics implied by the concept of evolutionary stability in a special limiting case. When mutations cannot occur simultaneously in the behavioral and physical components of the phenotype, mutations in the behavioural component of the phenotype occur more rapidly than those in the physical component, and selection is much faster than mutation in both components, the resulting separation of time scales has two important consequences. First, the behavioural component $\textbf{p}_R$ of the resident phenotype can only briefly differ from a $\textbf{p}_{m_R}$ evolutionary stable strategy (ESS) of the *A* matrix game played in the resident population characterized by $m_R$. The evolutionary stability of $\textbf{p}_{m_R}$ means that for any $\textbf{q}\ne \textbf{p}_{m_R}$ behaviour either i) $\textbf{p}_{m_R} A\textbf{p}_{m_R}>\textbf{q}A\textbf{p}_{m_R}$, or ii) if $\textbf{p}_{m_R} A\textbf{p}_{m_R}=\textbf{q}A\textbf{p}_{m_R}$, then $\textbf{p}_{m_R} A\textbf{q}>\textbf{q}A\textbf{q}$ holds^6^. Second, the behavioural component does not immediately adapt to the rare mutations in the physical component, but rather only with delay, in case a mutation prevails. This means that in the $W_M (\varepsilon )>W_R (\varepsilon )$ condition for substitution $\textbf{p}_M=\textbf{p}_R$, and only once the new $m_M$ trait component replaces the originally resident $m_R$ trait does selection fit the behavioural component to an ESS of the *D* matrix game.

As a result, evolution in this limiting case unfolds in a series of two-part steps:

1. Trait evolution: In a resident population of $(\textbf{p}_{m_R},m_R)$ phenotype individuals, where $\textbf{p}_{m_R}$ is the ESS of the A payoff matrix defined by the $m_R$ physical trait, a successful $(\textbf{p}_{m_R},m_M)$ mutant, which only differs in its physical trait, appears. This $(\textbf{p}_{m_R},m_M)$ mutant spreads through the population and replaces the resident $(\textbf{p}_{m_R},m_R)$ phenotype; the population now consists only of $(\textbf{p}_{m_R},m_M)$ individuals. $(\textbf{p}_{m_R},m_R)\rightarrow (\textbf{p}_{m_R},m_M)$
2. Behavioural adaptation: The $\textbf{p}_{m_R}$ behavioural strategy component of the original resident phenotype is quickly replaced in the population by the $\textbf{p}_{m_M}$ ESS belonging to the $m_M$ physical trait; this may happen in a series of rapid successive replacements; $(\textbf{p}_{m_M},m_M)$ becomes the new resident phenotype. $(\textbf{p}_{m_R},m_M)\rightarrow [(\textbf{p}_1,m_M)\rightarrow (\textbf{p}_2,m_M)\rightarrow \cdots \rightarrow (\textbf{p}_{n-1},m_M)]\rightarrow (\textbf{p}_{m_M},m_M)$

This separation of timescales highlights the composite nature of our interaction model. On the one hand, it is a matrix game whose strategies we identify as behaviours; however, the payoff entries of this matrix game are determined by another evolvable parameter of the players, a physical trait. On the other hand, it is a trait game of continuous strategies that has a multidimensional payoff expressed via four payoff functions; however, how the players actually weigh the contributions of these payoff functions is determined by another evolvable parameter of the players, a behaviour. So, we can think of the whole game as a continuous trait game embedded into a matrix game or, vice versa, as a matrix game embedded into a continuous trait game. The trait evolution step of the evolutionary process does not affect player behaviour, so it is better described by the latter interpretation. Conversely, the behavioural adaptation step leaves the traits unchanged, so it fits the former interpretation.

Since mutations in the physical trait occur rarely compared to selection in the behavioural strategy, the trait evolution part of the evolutionary steps takes longer and sets the overall timescale of the evolutionary process. The resulting evolutionary path of the population is the $(\textbf{p}_{m_{R_0}},m_{R_0})$, $(\textbf{p}_{m_{R_1}},m_{R_1})$, $(\textbf{p}_{m_{R_2}},m_{R_2})$, ...series of the successive resident populations the evolutionary steps visit. We will refer to the union of all possible evolutionary paths starting from different initial resident populations in our model as the evolutionary trajectory.

When, moreover, mutations in the physical trait component occur in small steps, that is, $|m_M-m_R|<<1$, then it follows from the $W_M(\varepsilon )>W_R(\varepsilon )$ condition for the $(\textbf{p}_{m_R},m_R)\rightarrow (\textbf{p}_{m_R},m_M)$ substitution – via Taylor expansion – that the direction of the evolution of the physical trait component in a monomorphic population of phenotype $(\textbf{p},m)$ is determined by the sign of the quantity

12 $$\begin{aligned} \Delta (m)=p^2 \partial _1 a_{SS} (m,m)+p(1-p) \partial _1 a_{SC} (m,m)+p(1-p) \partial _1 a_{CS} (m,m)+(1-p)^2 \partial _1 a_{CC} (m,m), \end{aligned}$$

where $\partial _1$ denotes partial differentiation with respect to the first physical trait variable of the payoff functions. Namely, when $\Delta (m)>0$, *m* increases; when $\Delta (m)<0$, *m* decreases; and when $\Delta (m)=0$, contributions from higher-order terms of the expansion decide whether *m* is a stationary point of the evolutionary process. Note that this result does not depend on the specific form of the payoff functions or, consequently, on the details of how the players interact.

During the evolutionary process, the behavioural component of the successive $(\textbf{p},m)$ resident populations is always the ESS of the matrix game played in the population. As it is known in the theory of matrix games, what this ESS is for any given physical trait *m* is decided by the signs of

13 $$\begin{aligned} x(m)&=a_{SS} (m,m)-a_{CS} (m,m),\end{aligned}$$

14 $$\begin{aligned} y(m)&=a_{CC} (m,m)-a_{SC} (m,m), \end{aligned}$$

the incentives to match an opposing player’s strategy [i.e., *x*(*m*) is positive if *S* is a better response to *S* than *C*, *y*(*m*) is negative if *S* is a better response to *C* than *C*]. For details on how this follows from the Bishop–Cannings theorem^49^, see section SI.1 of the Supplementary Information online. Whenever the ESS is a pure strategy, that is, $p=1$ or $p=0$, it is more instructive to classify games based on whether they constitute a social dilemma. Whether a game with a single pure-strategy ESS poses a social dilemma is decided by the sign of

15 $$\begin{aligned} d(m,m)=a_{SS}(m,m)-a_{CC}(m,m), \end{aligned}$$

the quantity that measures whether the players could improve their payoff by switching away from the pure ESS strategy, and thus determines whether the individual interests of the players are at odds with their interests as a group.

**Fig. 1:**
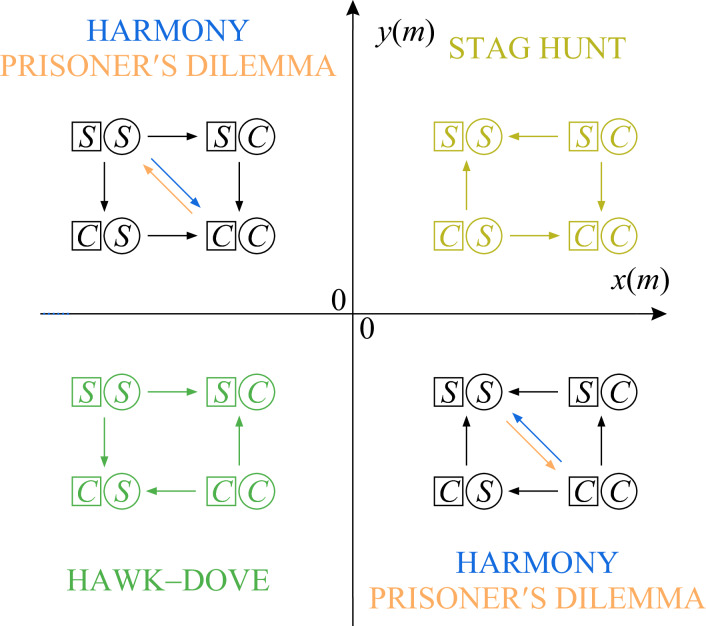
The classification map of symmetric $2\times 2$ matrix game types. The four quadrants of the *x*–*y* plane [see Eqs. (13), (14) and section SI.1 of the Supplementary Information online] correspond to the four game classes formed by games with different ESSs: Counter-clockwise, starting from the top right, bistable stag hunt games (yellow) are located in quadrant I, games in which the second pure strategy *C* is the only ESS in quadrant II, mixed-strategy ESS hawk–dove games (green) in quadrant III, and games in which the first pure strategy *S* is the only ESS in quadrant IV. Games along the boundaries separating the quadrants belong to the neighbouring single-pure-ESS class. It is often more instructive to reclassify games in the single-pure-ESS classes based on whether they constitute a social dilemma, which is decided by the sign of the difference of the diagonal payoff entries *d*(*m*, *m*) defined in Eq. (15): those games that pose a social dilemma are called prisoner’s dilemmas (orange), those games that do not are called harmony games (blue). The game classes are illustrated by correspondingly coloured flow graphs, which show the payoff change incentives [see matrix A in Eq. (10)] of unilateral strategy changes (vertical and horizontal arrows for the ‘square’ and ‘circle’ players, respectively) and the higher diagonal payoff entry (diagonal arrows) for single-pure-ESS games. An interaction model can only realize a game type if its evolutionary trajectory – that is, the curve traced by the resident payoff matrix *A* on this map as *m* runs through its possible values – visits the corresponding quadrant. For a concrete example, see Fig. 3. (The different colours should become different greys in greyscale prints).

As *m* runs through its possible values, the *x*(*m*) and *y*(*m*) incentive coordinates of the corresponding resident populations trace a representation of the evolutionary trajectory on the map shown in Fig. 1. This means that when trait evolution occurs in small steps and the *x*(*m*) and *y*(*m*) incentives are continuous in *m*, the resident game class significantly limits the outcome the behavioural adaptation part of an evolutionary step can typically have: The behaviour is only expected to incrementally change if the resident population plays a hawk–dove game [$x(m)<0$ and $y(m)<0$]. Sudden, jump-like changes in behaviour from one pure strategy to the other are also possible, but only if the ESS played in a monomorphic stag hunt population loses its evolutionary stability [$x(m)=0$ and $y(m)>0$ or $x(m)>0$ and $y(m)=0$] or if the evolutionary path goes through the zero-incentive [$x(m)=0$, $y(m)=0$] game.

### An illustrative numerical example

Because of its rather general nature, the model of hunting in pairs we introduced above is quite complex even in its simplest forms (for more details, see section SI.2 of the Supplementary Information online); it is defined by four non-negative functions (prey size *k*, appetite *b*, strategy costs $c_S$ and $c_C$) and four success probabilities ($w_{CC}$, $w_{CS}$, $w_{SC}$, and $w_{SS}$). Hence, we will not attempt to provide here a comprehensive treatment of the rich variety of behaviour our proposed model framework may exhibit. Instead, we present an arbitrarily chosen concrete numerical example relevant to the solution of social dilemmas that, we think, illustrates well the new possibilities the trait-mediated variability of payoff matrices opens up for the modelling of evolutionary processes.

**Fig. 2:**
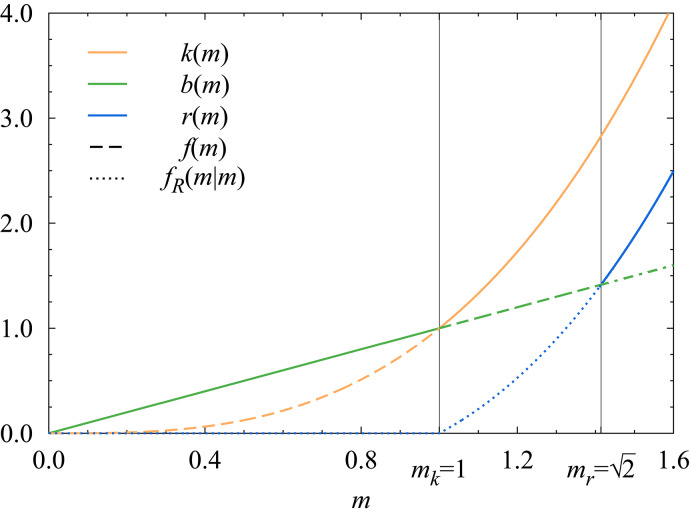
Graphs of the *k*(*m*) prey size (orange), the *b*(*m*) appetite (green), the *r*(*m*) leftover (blue), and the *f*(*m*) and $f_R(m|m)$ intake (dashed, dotted, and dash-dotted when equal) functions in the concrete example discussed in this section.

Let the defining functions and parameters of the model be the following:

16 $$\begin{aligned} k(m_1)=m_1^3,\quad b(m_1)=m_1, \end{aligned}$$

that is, let the appetite of a predator be sated by a prey of equal size and let the size of the capturable prey grow much faster above unit size, with the cube of predator size $m_1\ge 0$. The graphs of the resulting leftover and intake functions are shown in Fig. 2.

17 $$\begin{aligned} c_S (m_1)=\gamma _S m_1=0.1\cdot m_1,\quad c_C (m_1 )=\gamma _C m_1=0.2\cdot m_1, \end{aligned}$$

which means that the fitness costs of both hunting strategies are proportional to predator size with proportionality factors $\gamma _S$ and $\gamma _C$; openly chasing the herd is riskier than stalking ($\gamma _C>\gamma _S$).

18 $$\begin{aligned} w_{SS}=0.1,\quad w_{CS}=0.2,\quad w_{CC}=0.4,\quad w_{SC}=0.425, \end{aligned}$$

whereby the more active chasing strategy has a higher success rate when the hunting partner stalks ($w_{CS}>w_{SS}$) and, conversely, the more opportunistic stalking strategy is more likely to result in a kill when the hunting partner chases ($w_{SC}>w_{CC}$); furthermore, the disarray caused by a chasing partner also increases the chances of success ($w_{SC}>w_{SS}$ and $w_{CC}>w_{CS}$).

**Fig. 3:**
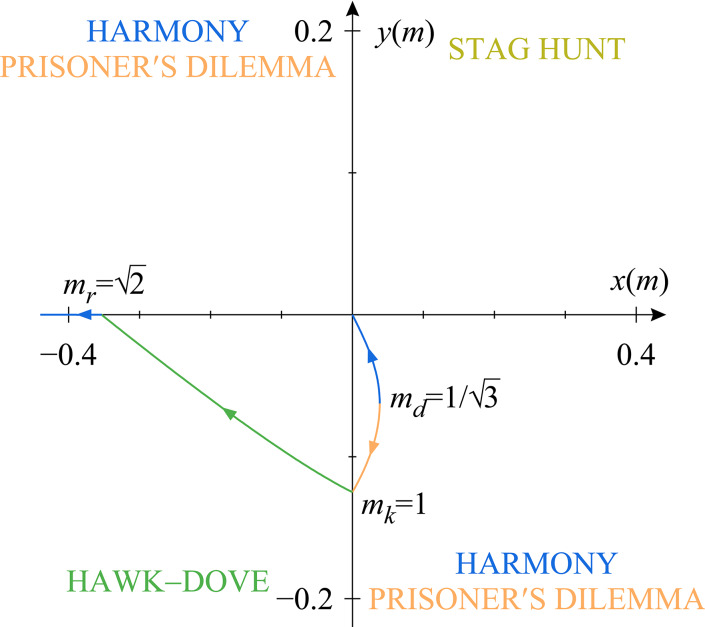
The evolutionary trajectory of the numerical example model defined by Eqs. (16)–(18) plotted with a solid line on the classification map of symmetric $2\times 2$ matrix game types. The classification is explained in section SI.1 of the Supplementary Information online and the caption of Fig. 1. The colours of the line indicate game type changes – that is, changes in which pure strategies are involved in the $\textbf{p}$ behaviour of the resident predator population – along the evolutionary trajectory, in accordance with the results shown in Fig. 4. The arrowheads along the evolutionary trajectory point towards the direction of evolution resulting from the adaptation of predator size *m* and, consequently, the payoff differences *x*(*m*) and *y*(*m*). The solid line demonstrates that the evolutionary path of predator populations initially playing a prisoner’s dilemma leads to a harmony game favouring the chasing strategy *C* via a series of hawk–dove games in our numerical example: Starting from an initial population with $m_k=1>m_0>m_d=1/\sqrt{3}$, the physical trait *m* increases. For a while, stalking is a best response to both stalking and chasing, but mutual chasing has a higher payoff than mutual stalking, the matrix game played in the population is a prisoner’s dilemma. As the trait crosses $m_k=1$, the trait for which prey size and appetite are equal, *x*(*m*) turns negative, *C* becomes the best response to *S*, and the population matrix game changes into a hawk–dove game. As *m* keeps increasing, *p* decreases, the probability of stalking incrementally becomes lower and lower, until it vanishes at $m_r=\sqrt{2}$, the trait for which a single captured prey satisfies the appetite of two predators. Then $y(m)=0$ and *S* ceases to be the better answer to *C*, too. While *m* keeps increasing, the behaviour no longer changes, the players always chase, and the game belongs to the harmony class.

Plotting the location of all possible resident populations on the *x*–*y* incentive plane defined by Eqs. (13) and (14) (see Fig. 3), we find that the probability of choosing to stalk in the ESS is: $p=1$ if $0<m\le m_k=1$; $0<p<1$ characteristic of hawk–dove games if $m_k<m<m_r=\sqrt{2}$; and $p=0$ if $m_r\le m$. It turns out (see section SI.2 of the Supplementary Information online) that in the chasing, $p=0$, $m_r\le m$ regime $a_{SS}(m,m)<a_{CC}(m,m)$, which means that individual and group interests are aligned and the game is a harmony game. On the other hand, the stalking, $p=1$ regime is not uniform in this regard. For $0<m<m_d=1/\sqrt{3}$, $a_{SS}(m,m)>a_{CC}(m,m)$, so these games are also harmony games; however, when $m_d<m<m_k$, this changes and $a_{SS}(m,m)<a_{CC}(m,m)$, which in these cases means that the group interests indicated by the higher diagonal payoff function are at odds with the individual interests indicated by the ESS, so these games belong to the prisoner’s dilemma class.

**Fig. 4:**
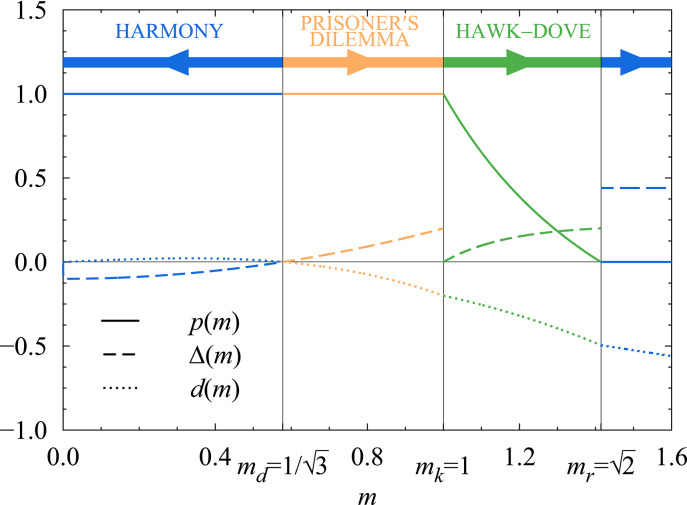
The ESS mixing parameter *p*(*m*) (solid line) and the determinants of the direction of evolution $\Delta (m)$ [see Eq. (12), dashed line] and whether a single-pure-ESS game is a social dilemma *d*(*m*, *m*) [see Eq. (15), dotted line] plotted against predator size *m* along the evolutionary trajectory of the numerical example discussed in this section. The thick arrows at the top show the direction of evolution, as indicated by the sign of $\Delta (m)$. The graphs are coloured in accordance with Fig. 3 and the game classification scheme presented in more detail in section SI.1 of the Supplementary Information online.

The *x*(*m*)–*y*(*m*) parametric curve in itself only describes the evolutionary trajectory of the model, but it says nothing about which parts of it and how the evolutionary process actually visits. As mentioned above, the direction of evolution is determined by the sign of $\Delta (m)$. In our example (see Fig. 4), $\Delta (m)$ changes sign in just a single point at $m_0=1/\sqrt{3}$ ($m_0=m_d$ is merely a coincidence, not a general feature); $\Delta (m)$ is negative if *m* is below, while $\Delta (m)$ is positive if *m* is above this threshold.

As a result, the evolutionary dynamics unfolds in one of five ways depending on the initial $m_i$ predator size: If $m_i<m_0$, then predator size decreases during evolution, while the game played in the population remains a harmony game throughout. Conversely, if $m_i>m_0$, evolution steadily increases predator size. It can also change the behavioural component: The game played in the population is a $p=1$ prisoner’s dilemma as long as $m_0<m\le m_k$, an *m*-dependent mixed-strategy hawk–dove game while $m_k<m<m_r$, and a $p=0$ harmony game in the $m_r\le m$ regime. When $m_0<m_i\le m_k$, the evolution spans all three regions and thus constitutes a gradual transition that abolishes the initial social dilemma. The special case of $m_i=m_0$ stays unchanged, as it is an unstable stationary point of the evolutionary dynamics.

We can interpret these evolutionary paths as follows: When predators are too small, hunting is unsustainable, there is no conflict of interest between pairs and individuals, and fitness can be increased by decreasing the cost associated with hunting, which is achieved by choosing the more cautious stalking strategy and a further decrease in predator size. This cycle could be directly interpreted as a form of extinction, but seeing it as a gradual reallocation of the resource holding potential towards other food sources provides a perhaps more flexible explanation.

At larger predator sizes, however, an incremental increase in predator size decreases the gap between intake and appetite as the increase in the spoils of a successful hunt start to outweigh the costs of the increase in risk, thus leading to a net increase in fitness, while mutual chasing becomes lucrative enough to turn the game into a prisoner’s dilemma. Once predator size becomes large enough, a successful hunt not only satisfies the hunter but also produces a byproduct: The possibility of scavenging leftovers effectively lowers the cost of failed hunts, thus disincentivizing stalking while the partner also stalks, allowing both hunting strategies to be present in the population. When captured prey become large enough, any successful hunt satisfies both hunters, and stalking no longer provides a fitness advantage over a chasing partner, which establishes mutual cooperation in chasing the herd.

## Discussion

In the present article, we proposed a coevolutionary matrix game model in which the payoffs do not only depend on the strategies the interacting players choose but also some other evolvable trait. One of our aims was to find a mechanism for supporting cooperation in social dilemma situations that hews even closer to classical evolutionary game theory than the numerous available explanations mentioned in the introduction. We would like to reiterate that the model we discuss in this paper is not intended to be a full-fledged treatment of any realistic ecological situation but rather an introductory illustration of the capabilities of what we believe is a natural yet unexplored extension of a classical approach to describing evolutionary processes. We hope that the deliberate parsimony of our analysis offers the mathematical and conceptual clarity that will allow our model framework to serve as a foundation for future, more empirically-driven applications.

Our model maintains the core assumptions underlying the concept of evolutionary stability introduced by Maynard Smith and Price^6^. It considers an asexually reproducing monomorphic population occasionally perturbed by mutations. The population is well-mixed, the specimens of the resident and mutant phenotypes interact with each other with a frequency that reflects the makeup of the population – inhomogeneities in the density of the population are disregarded. Neither the available strategy choices nor the rules that govern the dynamics in our model offer any external options not inherent to the interaction or the interpretation of the payoffs as reproductive fitness – the players have no memory of their opponents’ previous choices. Even the consequences of our result that say that evolution in our model can change the very character of the interactions do not necessarily have to be immediately obvious to an outside observer: For example, if it is only some internal, hidden, or otherwise hard to ascertain component of the payoffs (such as the costs associated with playing certain strategies) that actually depends on the evolving trait, then the changes in the interaction might go unnoticed next to the resulting changes in player behaviour.

Our model of hunting in pairs relies on the prey animals living in a group and the predators having to attack the herd. This condition ensures that the otherwise solitary hunters end up hunting together from time to time, and this is what ultimately generates the game-theoretical conflict. If hunting in pairs is more fruitful than hunting alone, then it is reasonable to assume that the predators will adapt and start to tolerate being near each other, which could eventually lead to group formation. These groups could be capable of capturing even larger prey, which could bring further advantage to group members.

The assumption that the mass of the prey captured by a single predator grows faster than the amount of flesh the predator can consume seems to be of key importance for establishing cooperation. In our concrete example, the higher costs associated with the riskier chasing option causes stalking to be the preferred behaviour for smaller predator sizes, despite its lower success rate. If the predators are too small, the available prey are not large enough to sustain the predators, thus there is no social dilemma, and evolution further shrinks the predators to lower risk-related costs. For larger predator sizes, however, the gap between predator appetite and prey size becomes smaller, and chasing becomes lucrative enough to turn the game into a prisoner’s dilemma. In this case, predator size starts to increase, as it allows predators to kill larger prey, which further decreases the gap and increases intake. Once prey size becomes large enough, successful predators are sated by their prey, and unsuccessful predators have access to leftovers, further mitigating the risks of chasing and lowering the incentive to stalk; the game eventually becomes a hawk–dove game, which allows stalkers and chasers to sustainably coexist. Once killed prey become large enough to fully feed two predators, group interests and individual interests are no longer distinguishable, so the game becomes a harmony game.

While the analysis of the previous paragraph does rely on some of the specificities of our concrete example (e.g., the model does not always end up being a harmony game that prefers chasing in the limit of large predator sizes), our core results about how matrix games with a variable payoff-determining trait evolve in monomorphic populations in the limit of rapid selection and moderately frequent or rare mutations are quite general: They do not depend on the details of the interaction and the specific form of the payoff functions. Furthermore, our results should readily generalise to model setups with more available behavioural strategies or more payoff-determining trait variables.

In a future study, we intend to explore how the dynamics changes if the assumption of rapid selection is relaxed. In this case, the time scales do not separate and the different phenotype components evolve simultaneously, which we expect to open up new evolutionary trajectories.

Future applications of our theoretical modelling framework, if they are to have real descriptive and predictive power, will need to conduct a more thorough analysis than the deliberately simplified pedagogical example we presented in this paper. Among other things, they will certainly have to address the following issues: First, the choice of the specific payoff functions and their parameters will require stronger empirical underpinnings. More realistic interaction models tend to be more complex with more parameters, which may pose additional technical difficulties. Furthermore, currently available data may prove insufficient for inferring certain model parameters. Second, the robustness and validity of the results will need to be checked. Sensitivity analysis and extracting actually measurable quantities for comparison with field data may become more complicated as the number of parameters increases. Presenting the results in a truly informative way may also be its own challenge. Third, extending the concept of evolutionary stability to polymorphic populations will be necessary. Analytic treatments may be technically demanding; using simulation approaches may be inevitable.

As we mentioned above, hunting in lions only served as an inspiration for our model. One of the reasons behind this is that – unlike the predators in our model – real lions reproduce sexually and the females that hunt together are related^39^. This means that the analysis of the evolution of lions requires population genetic models that take kinship into account. We note that cooperator and defector phenotypes can coexist within the framework of Haldane’s familial selection model^50–53^, but the consequences of the evolution of trait-dependent payoff matrices have yet to be explored in this context.

## Supplementary Information

Supplementary Information.

## Data Availability

All data generated or analysed during this study are included in this published article and its supplementary information files.
