# Supplementary material for: Evolutionary path from a prisoner’s dilemma to a harmony game via hawk–dove games
Source: Sci Rep. 2026 Apr 13;16:17207. doi: 10.1038/s41598-026-47565-9 (PMC13233841; doi:10.1038/s41598-026-47565-9)
Supplement: Supplementary file 1 — Supplementary Information. [file 41598_2026_47565_MOESM1_ESM.pdf]

Supplementary Information

to *Evolutionary path from a prisoner's dilemma to a harmony game via hawk–dove games*

by Balázs Király, Tamás Varga, and József Garay

SI.1 Evolutionarily stable strategies (ESS) in a resident monomorphic population

Using the notation introduced in the main text, the $\mathbf{p}_m$ evolutionarily stable strategies of a resident monomorphic population of physical trait m are defined by the following two-part condition holding true for all other $\mathbf{q} \neq \mathbf{p}_m$ mixed strategies (see Ref. [6] of the main text):

i $\mathbf{p}_m A \mathbf{p}_m > \mathbf{q} A \mathbf{p}_m$, or

ii if $\mathbf{p}_m A \mathbf{p}_m = \mathbf{q} A \mathbf{p}_m$, then $\mathbf{p}_m A \mathbf{q} > \mathbf{q} A \mathbf{q}$.

According to the Bishop–Cannings theorem (see Ref. [46] of the main text), the ESSs of a matrix game with two pure strategies are either one or both of those two pure strategies or a unique mix of them. In the latter case, the payoffs of the pure strategies against this unique mixed strategy are equal to each other. With this in mind, it is easy to check that depending on the entries of the 2×2 payoff matrix A there are just four possible ESS configurations:

I When $x(m) > 0$ and $y(m) > 0$, then both $(1, 0)$ and $(0, 1)$ are ESSs, so the game is of the stag hunt type.

II When $x(m) \leq 0$ and $y(m) \geq 0$ and $(x(m), y(m)) \neq (0, 0)$, then only $(0, 1)$ is an ESS.

III When $x(m) < 0$ and $y(m) < 0$, then the game has a mixed ESS, $\left(\frac{y(m)}{x(m)+y(m)}, \frac{x(m)}{x(m)+y(m)}\right)$, which is a defining characteristic of hawk–dove games.

IV When $x(m) \geq 0$ and $y(m) \leq 0$ and $(x(m), y(m)) \neq (0,0)$, then only $(1,0)$ is an ESS.

Notice that these four game classes occupy the four quadrants of the x–y plane. (See Figure 1 of the main text.) [If $(x(m), y(m)) = (0,0)$, then every strategy is a Nash a equilibrium, so no ESS exists. Otherwise, an ESS always exists.]

It is often more instructive to classify games that have only one pure ESS on the basis of whether they constitute a social dilemma instead of which of their pure strategies happens to be their ESS:

A When $x(m) \leq 0$ and $y(m) \geq 0$ and $a_{SS}(m,m) > a_{CC}(m,m)$ or $x(m) \geq 0$ and $y(m) \leq 0$ and $a_{SS}(m,m) < a_{CC}(m,m)$, then the interests of the individuals – as indicated by the ESS – and the interests of the group – as indicated by the higher diagonal payoff entry – are at odds and the game poses a social dilemma; these games belong to the prisoner's dilemma class.

B When $x(m) \leq 0$ and $y(m) \geq 0$ and $a_{SS}(m,m) \leq a_{CC}(m,m)$ or $x(m) \geq 0$ and $y(m) \leq 0$ and $a_{SS}(m,m) \geq a_{CC}(m,m)$, then there is no conflict between individual and group interests and the game poses no social dilemma; these games belong to the harmony class.

The duality of the ESS in the I stag hunt regime and especially how it affects the evolutionary trajectory raises a number of questions whose answers lie beyond the scope of the present paper. We hope to return to these questions in a future study.

SI.2 Some motivation for our numerical example

If we directly interpret the m_1 predator size, $k(m_1)$ captured prey size, and $b(m_1)$ appetite quantities as masses in our model of hunting in pairs, then it is reasonable to assume that

a) $m_1 \geq 0$, $k(m_1) \geq 0$, $b(m_1) \geq 0$;

b) both $k(m_1)$ and $b(m_1)$ grow monotonously with increasing m_1;

c) $k(0) = 0$ and $b(0) = 0$.

Making a distinction between the $k(m_1)$ prey size, the $b(m_1)$ appetite, the $r(m_1)$ leftover, and the $f(m_1)$ intake only really makes sense if no two of these quantities are obviously equal to each other. This is clearly the case when for small enough values of m_1 it is $b(m_1)$, while for large enough values of m_1 it is $k(m_1)$ that grows faster as m_1 is increased, since this means that predators below a threshold size are not satisfied by the prey they can capture [$k(m_1) < b(m_1)$], whereas predators above a (possibly different) threshold size are guaranteed to leave leftovers for an unsuccessful partner [$r(m_1) = k(m_1) - b(m_1) > 0$].

In the simplest variants of this model there is a single $m_k > 0$ threshold, which satisfies the following three conditions: $b(0 < m_1 < m_k) > k(0 < m_1 < m_k)$, $b(m_k) = k(m_k)$, $b(m_k < m_1) < k(m_k < m_1)$. In other words there is a single "optimal" m_k predator size for which the size of the captured prey exactly satisfies the predator's appetite; smaller predators can only catch "small prey" that do not fully satisfy the predator; larger predators can only catch "large prey" that they cannot fully consume.

Our choices of cubic $k(m_1) = m_1^3$ prey size and linear $b(m_1) = m_1$ appetite growth in our example from the main text clearly satisfy these conditions. If we further assume that the cost functions also linearly increase with predator size, it follows that along the $m_1 = m_2 = m$ evolutionary trajectory the $x(m)$ and $y(m)$ functions, similarly to the payoff functions, take the form

$$x(m) = \begin{cases} \alpha_x m^3 + \beta m & \text{if } m \leq m_k \\ (\alpha_x + \delta_x)m^3 + \quad (\beta - \delta_x)m & \text{if } m_k < m < m_r \\ (2\alpha_x + \delta_x + \beta)m & \text{if } m_r \leq m \end{cases}, \tag{SI.1}$$

$$y(m) = \begin{cases} \alpha_y m^3 - \beta m & \text{if } m \leq m_k \\ (\alpha_y + \delta_y) m^3 + (-\beta - \delta_y) m & \text{if } m_k < m < m_r \\ (2\alpha_y + \delta_y - \beta) m & \text{if } m_r \leq m \end{cases}, \tag{SI.2}$$

where we introduced the following shorthand notations:

$$\alpha_x = w_{SS} - w_{CS}, \quad \delta_x = w_{CS} - w_{SC} + w_{SC}w_{CS} - w_{SS}^2, \tag{SI.3}$$

$$\alpha_y = w_{CC} - w_{SC}, \quad \delta_y = w_{SC} - w_{CS} + w_{SC}w_{CS} - w_{CC}^2, \tag{SI.4}$$

$$\beta = \gamma_C - \gamma_S, \tag{SI.5}$$

m_k is the size of the predator whose prey perfectly satisfies its appetite $[k(m_k) = b(m_k)]$, and m_r is the size of the predator whose prey fills both itself and an unsuccessful partner of the same size $[k(m_r) = 2b(m_r)]$. Finally, we note two general properties of this model that informed our choice of the example presented in the main text:

In the $m \to 0$ limit, terms proportional to m^3 become negligibly small and $x(m) \approx \beta m$ and $y(m) \approx -\beta m$ whenever $\beta \neq 0$. This means that the evolutionary trajectory tends to the $y = -x$ line, and thus falls into either the II $\mathbf{p}_m = (0, 1)$ or the IV $\mathbf{p}_m = (1, 0)$ single-pure-ESS quadrant of the x–y plane for small values of m. Similar analyses of $a_{CC}(m, m) - a_{SS}(m, m)$, which decides whether the game is a social dilemma, and $\partial_1 a_{CC}(m, m)$ and $\partial_1 a_{SS}(m, m)$, which decide the direction of evolution via $\Delta(m)$, reveal that in the $m \to 0$ limit the game played in the resident population is a harmony game that favours the less costly hunting strategy and predator size decreases during evolution, regardless of what values the w success rates take.

When m is not too small, however, the cubic terms are no longer negligible, which may allow them to change the signs of $x(m)$ and $y(m)$ and, as a result, which class the game belongs to. Keeping in mind that the predator size m is not negative, we find that the signs of $x(m)$ and $y(m)$ can each change at most once on both the $0 < m \leq m_k$

and the $m_k < m \leq m_r$ intervals, respectively at m_{x0}, m_{x00}, m_{y0}, and m_{y00}, where $m_{x0}^2 = -\frac{\beta}{\alpha_x}$, $m_{x00}^2 = -\frac{\beta-\delta_x}{\alpha_x+\delta_x}$, $m_{y0}^2 = \frac{\beta}{\alpha_y}$, $m_{y00}^2 = \frac{\beta+\delta_y}{\alpha_y+\delta_y}$, provided, of course, that these points actually fall into the appropriate intervals. $a_{CC}(m,m) - a_{SS}(m,m)$ behaves similarly. Consequently, the evolutionary trajectory can reach any quadrant of the x–y plane as m runs over the $[0, m_r]$ interval, that is, all four game classes defined by the different possible ESS configurations can already be realized even in this restricted model family characterized by cubic prey size and linear appetite and cost functions. When $m_r < m$, then the evolutionary trajectory traces a half-line pointing away from the origin of the x–y plane as m increases, so the type of the game played in the game remains the same as that of the one at m_r.

Note that the above analysis only considers the possible evolutionary trajectory but not the actual direction of evolution, which is determined by the sign of $\Delta(m)$.
